# Increased Risk of Fracture Among Patients With Iron Overload: A Population-based Matched Cohort Study

**DOI:** 10.1210/clinem/dgae807

**Published:** 2024-11-18

**Authors:** Andrea Michelle Burden, Adrian Martinez-De la Torre, Theresa Burkard, Maria Immoos, Lorenz Christian Hofbauer, Andrea Ulrike Steinbicker, Martina Rauner

**Affiliations:** Pharmacoepidemiology Group, Institute of Pharmaceutical Sciences, Department of Chemistry and Applied Biosciences, ETH Zurich, 8093 Zurich, Switzerland; Leslie Dan Faculty of Pharmacy, University of Toronto, Toronto, ON M5S 3M2, Canada; Pharmacoepidemiology Group, Institute of Pharmaceutical Sciences, Department of Chemistry and Applied Biosciences, ETH Zurich, 8093 Zurich, Switzerland; Pharmacoepidemiology Group, Institute of Pharmaceutical Sciences, Department of Chemistry and Applied Biosciences, ETH Zurich, 8093 Zurich, Switzerland; Pharmacoepidemiology Group, Institute of Pharmaceutical Sciences, Department of Chemistry and Applied Biosciences, ETH Zurich, 8093 Zurich, Switzerland; Division of Endocrinology, Diabetes, and Bone Diseases & University Center for Healthy Ageing, Department of Medicine III, Technische Universität Dresden, 01307 Dresden, Germany; Department of Anaesthesiology, Intensive Care Medicine and Pain Therapy, Goethe University Frankfurt, University Hospital Frankfurt, 60596 Frankfurt, Germany; Division of Endocrinology, Diabetes, and Bone Diseases & University Center for Healthy Ageing, Department of Medicine III, Technische Universität Dresden, 01307 Dresden, Germany

**Keywords:** osteoporosis, fracture, iron overload, red cells, iron, erythropoiesis

## Abstract

**Introduction:**

Iron overloading disorders are associated with decreased bone mineral density. However, evidence on fracture risk is scarce. Therefore, we evaluated the risk of fracture associated with iron overload disorders compared to matched controls.

**Methods:**

Using The Healthcare Improvement Network, a Cegedim database of UK general practice data, we identified patients >18 years with elevated iron (ferritin value >1000 µg/L) or an eligible diagnosis code for iron overloading disorders between 2010 and 2022. The first date of elevated iron or a diagnosis code defined the index date for iron overload patients, who were matched with up to 10 controls. Time-varying confounder-adjusted Cox proportional hazard models estimated the hazard ratios (HRs) and 95% confidence intervals. Analyses were stratified by osteoporotic fracture site (hip, vertebral, humerus, forearm) and evidence of elevated serum ferritin at baseline (ferritin >1000 µg/L), and sex.

**Results:**

We identified 20 264 eligible patients and 192 956 controls. Overall, there was a 55% increased risk of any fracture among iron overload patients (HR 1.55 [1.42-1.68]). Fracture risk was increased at all sites, with the highest risk observed for vertebral fractures (HR 1.97 [1.63-2.10]). Patients with ferritin >1000 µg/L had a 91% increased risk of any fracture (HR 1.91 [1.73-2.16]) and a 2.5-fold increased risk of vertebral fractures (HR 2.51 [2.01-3.12]). There was no increased risk among patients without elevated serum ferritin at any site. Fracture risk was similar between sexes.

**Discussion:**

This large population-based cohort study found a 55% increased risk of fracture associated with iron overload. The risk was highest among patients with laboratory-confirmed iron overload, highlighting the importance for clinicians to consider initiating osteoporosis therapy in patients with serum ferritin >1000 µg/L to minimize fracture risk.

Osteoporotic fractures pose a major public health burden (1). In particular, hip fractures have devastating effects in terms of quality of life, morbidity, and mortality (2). While aging is the most common cause of osteoporosis, defined by reduced mineral bone density (BMD) and an impaired bone microarchitecture, diseases associated with a high iron loading have been shown to increase the risk for lower BMD and subsequent fracture risk.

Hereditary hemochromatosis (HH) is an inherited disorder of iron homeostasis leading to elevated blood iron levels (3). Additionally, thalassemia major and sickle cell anemia are inherited blood disorders that can cause an accumulation of iron in the human body, which can be deposited in the liver, pancreas, heart, or joints, similar to that seen in HH (3). Iron-loading anemias are characterized by high serum iron, transferrin saturation, and ferritin values as well as hemosiderin deposits in parenchymal cells and reticuloendothelial tissue with or without organ dysfunction. While distinct in their etiology and presentation, HH and iron-loading anemias are associated with low BMD (4-8). Decreased BMD was found in >70% of sickle cell disease adult patients (9, 10) and in >60% of adult thalassemia patients (11). Osteoporosis was evident in 25% to 34% of patients with a genetic mutation of the *HFE* gene resulting in *HFE*-dependent HH, which is the most common form of HH (5, 6). More recent studies using data from the UK Biobank found that patients with homozygous mutation in *HFE* (C282Y) showed that males with *HFE*-dependent HH have double the risk of developing osteoporosis, although a nonsignificant effect was observed among females (8, 12).

While iron overload is associated with decreased BMD, imaging studies have demonstrated iron overload disorders may increase fracture risk due to the influence of both bone quantity and microarchitecture (13, 14). There is growing experimental and clinical evidence of the relationship between iron overload due to thalassemia major, hemochromatosis, or sickle cell anemia and bone homeostasis (15-18); however, the relationship to fracture risk remains understudied and inconsistent, particularly at different fracture sites (7, 11, 19). In the UK Biobank, only the risk of femoral fractures among male C282Y homozygotes demonstrated a significantly increased risk (12). Similarly, a previous case control of 306 HH cases and 304 controls found nonsignificant associations for vertebral, wrist, or hip fractures (7). However, when the HH cases were stratified by severity (defined as a serum ferritin >1000 µg/L), a significant 2.8- to 3.0-fold increased risk of vertebral and wrist fractures, respectively, was found (7). Along those lines, a study with 115 postmenopausal women identified elevated serum ferritin concentrations as an independent predicator of hip fractures (20). Thus, in this study we aimed to investigate the association between iron overload and osteoporotic fractures in a large UK-based cohort study.

## Methods

### Database

We used data from the IQVIA medical research database in the UK (UK IMRD), that incorporates data from The Healthcare Improvement Network, A Cegedim database (21). The UK IMRD is a dynamic longitudinal cohort, with over 18 million patient records from over 800 general practitioner practices, of which approximately 3 million patients and 370 practices are currently active and contribute data. Patients are included in the IMRD when they register with a practice that contributes data through The Healthcare Improvement Network or at the time in which the practice began contributing data, whichever comes first. Retrospective data on current and past patients are included at the time the patient or practice joins the IMRD data collection to obtain complete medical histories. Patient data are collected until the patient transfers out of a practice, the practice stops contributing data, or the patient dies.

Within the database, detailed information is available on patient characteristics (eg, year of birth, sex, practice registration date, practice deregistration date), medical conditions (eg, diagnoses with dates, referrals to hospitals, symptoms), medications (eg, drug name, formulation, date, strength, quantity), laboratory test results (eg, hemoglobin, ferritin), and other patient-level data (eg, smoking status, height, weight, alcohol use, pregnancy, birth, death dates). All medications are mapped according to the international Anatomical Therapeutic Codes (ATC) classification system and the British National Formulary code dictionary. For medical conditions, all diagnoses are coded according to the Read clinical code system, a comprehensive coding language with over 100 000 codes and comparable to the International Classification of Diseases system (22).

### Study Design and Study Population

We conducted a matched cohort study design using the UK IMRD database between January 1, 2010, and September 30, 2022. Iron overload patients were identified as those with a ferritin value >1000ug/L or an incident diagnosis of thalassemia major, sickle cell disease, or hemochromatosis. A complete code list used to identify eligible patients is provided in Supplementary Table S1 (23). Only patients and entries with valid flags were included. The date of the first record of a ferritin >1000ug/L or incident diagnosis code served as the index date for the iron overload patients. Of note, the laboratory parameter ferritin ≥200 µg/L for women and ≥300 µg/L for men together with a transferrin saturation ≥45% are clinical markers to screen for hemochromatosis—but they can also be caused by other circumstances such as receipt of blood transfusions, iron supplementation, or inflammation. The confirmation of the genetic inherited disease is the indicator to point to the diagnosis. Therefore, we looked for clear and very high ferritin levels and neglected those between 200/300 and 1000 µg/L.

We excluded patients who were aged <18 years at the index date, those who had an index date outside of the study period (ie, before January 1, 2010, or after December 31, 2020), those who had a recorded date of death or transfer out of the database before the index date, and those who had less than 1 year of database history before the index date. Additionally, we included a wash-out period of 1 year to identify new fractures following the index date; therefore patients with an osteoporotic fracture in the year prior to the index date were excluded. The code list of osteoporotic fractures is provided in Supplementary Table S2 (23). Additionally, patients who used iron supplementation within the 3 months prior to the index date were excluded because it may lead to temporary high iron levels.

We subsequently matched all patients with up to 10 control patients without increased iron levels or a diagnosis code throughout their observation period on age, sex, practice, duration on the database, and calendar date. Due to the low prevalence of iron overload or an eligible diagnosis code, matching was done without risk-set sampling. Controls were assigned the same index date as their matched iron overload patient. Eligible controls were those aged 18 years or older at the registration date in the practice, those who had a registration date that occurred before their date of death or transfer out of practice, those who had a registration date within the study period (January 1, 2010-December 31, 2020), or those who did not have a read code for an osteoporotic fracture prior to the assigned index date.

### Exposure of Interest

The primary exposure of interest was a record of iron overload, defined as a diagnosis of hemochromatosis, thalassemia, sickle cell disease, or serum ferritin value >1000 µg/L.

### Outcome of Interest and Follow-up

The primary outcome of interest was the first occurrence of an osteoporotic fracture following the index date. The validity of fracture codes in the UK General Practice data has previously been demonstrated (24). Osteoporotic fractures were defined as humerus, forearm, clinical vertebra, or hip (see Supplementary Table S2) (23). Patients were followed from the index date until the first occurrence of the outcome (ie, an eligible fracture), the end of patient record, the end of the study period (November 30, 2022), or death.

### Covariates

For all covariates, directed acyclic graphs were generated to confirm potential confounding vs mediating variables. We identified the following covariates at index date: age (continuous), sex, body mass index [body mass index (BMI): categorized based on World Health Organization definitions as underweight (BMI < 18.5), normal (BMI 18.5-24.9), overweight (BMI 25.0-29.9), and severely overweight (≥30.0)], smoking status (never, former, current, missing), and alcohol use (never, former, current, missing). These demographics and lifestyle factors were identified using an ever-before lookback window using the closest value to the index date and were evaluated for changes during follow-up (as time-varying covariates).

Additionally, using medical read codes, we identified a history of the following conditions at any time before the index date: blood-related disorders (ie, aplastic/hemolytic/pernicious anemia, leukemia), ulcers, neoplasms, nonmalignant skin cancer, inflammatory conditions (ie, rheumatoid arthritis, psoriasis, inflammatory bowel disease), bariatric surgery, thyroid disease, kidney disease, hypogonadism, liver disease, endocrine disorders, and prior fracture (any) > 365 days before the index date.

Using ATC codes, we assessed pain medications (eg, non-steroidal anti-inflammatory drugs) (ATC: M01A), systemic corticosteroids (ATC: H02), antihypertensives (ATC: C03, C07-09), osteoporosis medications (ATC: M05BA/B/X), noninsulin antidiabetic drugs (ATC: A10B), insulin, and contraceptives (ATC: G03A). Medications were identified if prescribed within the 6 months before the index date.

### Data Analysis

We described the patient characteristics at the index date as means and SDs or counts and proportions, as appropriate. A standardized mean difference (SMD) > 0.10 was used to identify significant differences between the patients with and without iron overload.

We described the crude incidence rates of fractures among patients with iron overload and separately among matched controls. Cox proportional hazard regression models were used to estimate the hazard ratio (HR) with 95% confidence intervals (CIs) for the risk of an osteoporotic fracture associated with iron overload compared to matched controls.

As a sensitivity analysis, we examined the impact of laboratory-confirmed iron overload on osteoporotic fracture risk. We stratified our cohort based on entering the cohort with a serum ferritin value >1000ug/L vs having a diagnosis code for thalassemia, hemochromatosis, or sickle cell anemia without laboratory evidence of iron overload. Additionally, in a post hoc analysis, we restricted the analysis to only patients with iron overload, whereby we stratified our cases by evidence of laboratory-confirmed iron overload (serum ferritin >1000ug/L) vs presence of a diagnosis code in the absence of laboratory-confirmed iron overload.

All analyses were stratified by fracture type (ie, hip, radius/ulna, clinical vertebral, humerus) and biologic sex.

Additionally, we included potential confounders that were unbalanced at baseline (SMD >0.10) or based on clinical consensus in all models.

Analyses were conducted using SAS software version 9.4. The protocol for the current study was reviewed and approved by the IMRD Scientific Research Council (SRC reference number 21SRC064). Additionally, the London-South East Research Ethics Committee (Ref 18/LO/0041) has approved the use of the IMRD for medical and public health research purposes. Further details can be found online at https://www.iqvia.com/locations/united-kingdom/information-for-members-of-the-public/medical-research-data#:∼:text=Ethics%20Approval. In accordance with data protection procedures, any count with a value <7 was suppressed in text and tables.

## Results

### Cohort Characteristics

**Figure 1.**
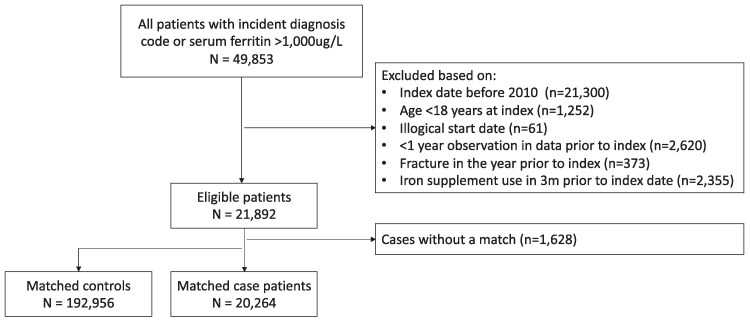
Patients flow diagram. Cases are those patients with an eligible diagnosis code for an iron overloading disorder [Supplementary Table S1 (23)] or a recorded serum ferritin value >1000 µg/L. The first date of the diagnosis or serum ferritin was considered the index date for inclusion into the study.

After exclusions as indicated in Fig. 1, we included 20 264 patients with iron overload and 192 956 matched controls (Fig. 1). The baseline characteristics are provided in Table 1. The mean age was 57 years in both groups, and approximately 40% were female (39.1% among patients and 42.3% among controls, SMD 0.06). Current alcohol consumption was higher among patients with iron overload compared to controls (21.1% vs 19.1%, respectively, SMD = 0.16). At baseline, patients with iron overload had a higher prevalence of malignant cancer diagnoses (23.0% vs 14.7%, SMD = 0.22) and liver disease (7.0% vs 1.9%, SMD = 0.25) when compared to controls without iron overload. Medication use in the 6 months prior to baseline was similar between the patients and controls, with the exception of the use of beta-blockers (15.6% among patients vs 12.4% among controls, SMD = 0.16), diuretics (25.5% among patients vs 17.7% among controls, SMD = 0.19), and oral glucocorticoids (14.7% among patients vs 9.0% among controls, SMD = 0.18).

**Table 1.** Baseline characteristics for patients and matched controls

	Patients with iron overload n = 20 264	Matched controls n = 192 956	SMD
Mean age (SD) [years]	57.8 (17.5)	57.7 (18.3)	0.01
Female (%)	7922 (39.1)	81 545 (42.3)	0.06
Mean follow-up (SD) [years]	4.7 (3.8)	4.2 (3.7)	0.13
Ferritin value available (%)	13 510 (66.7)	5613 (2.9)	1.80
Ferritin *μ*g/L [mean (SD)]	1731.4 (1440.4)	141.1 (222.4)	1.54
BMI (closest to index date)			
Mean (SD)	27.1 (5.9) (6.8)	27.3 (5.7)	0.03
Missing (%)	2554 (12.6)	28 206 (14.6)	0.06
Smoking status (closest to index date) (%)			
Current	4267 (21.1)	36 893 (19.1)	0.10
Former	5595 (27.6)	49 810 (25.8)	
Never	9251 (45.7)	97 465 (50.5)	
Missing	1151 (5.7)	8788 (4.6)	
Alcohol consumption (closest to index date) (%)			
Current	13 981 (69.0)	121 950 (63.2)	0.16
Former	762 (3.8)	5862 (3.0)	
Never	2689 (13.3)	35 339 (18.3)	
Missing	2832 (14.0)	29 805 (15.4)	
Covariates (ever before the index date) (%)			
Aplastic anemia	51 (0.3)	21 (0.0)	0.07
Cancer diagnosis	4660 (23.0)	28 279 (14.7)	0.22
Chronic kidney disease	538 (2.7)	2390 (1.2)	0.14
Hemolytic pernicious	236 (1.2)	1078 (0.6)	0.07
Inflammatory diseases*^a^*	472 (2.3)	3186 (1.7)	0.05
Thyroid disease	1346 (6.6)	12 097 (6.3)	0.02
Ulcers	727 (3.6)	5302 (2.7)	0.05
Pregnancy*^b^*	136 (0.7)	1238 (0.6)	0.00
Hypogonadism	31 (0.2)	196 (0.1)	0.01
Osteoporosis	710 (3.5)	5804 (3.0)	0.03
Liver disease	1420 (7.0)	3611 (1.9)	0.25
Endocrine disease	68 (0.3)	372 (0.2)	0.03
Prior fracture*^c^*	2162 (10.7)	17 733 (9.2)	0.05
Medication within 6 months before			
ACE inhibitors	5208 (25.7)	400 007 (20.7)	0.12
Antihypertensives	584 (2.9)	4145 (2.1)	0.05
Beta-blockers	3670 (15.6)	23 834 (12.4)	0.16
Calcium channel blockers	3159 (15.6)	25 397 (13.2)	0.07
Diuretics	5161 (25.5)	34 209 (17.7)	0.19
Oral glucocorticoids	2975 (14.7)	17 297 (9.0)	0.18
Bisphosphonates	701 (3.5)	5202 (2.7)	0.04
Noninsulin antidiabetic drugs	1246 (6.1)	12 742 (6.6)	0.02
Insulin	377 (1.9)	3425 (1.8)	0.01

Unless otherwise indicated, all values are the number and column proportions.

Abbreviations: ACE, angiotensin-converting enzyme; BMI, body mass index; SMD, standardized mean difference (calculated using Cohen's *d*).

^*a*^Includes a diagnosis of rheumatoid arthritis, psoriatic arthritis, inflammatory bowel disease, or other unspecified inflammatory disease.

^*b*^Pregnancy was identified based on diagnosis codes and identified within the 9 months prior to the index date.

^*c*^Prior fractures were those occurring >365 days before the index date.

### Risk of Osteoporotic Fracture Associated With Iron Overload

Table 2 presents the results from the primary analysis assessing the association between iron overload and osteoporotic fractures, stratified by fracture site and patient`s sex. Overall, patients with iron overload had a significantly increased risk of experiencing any osteoporotic fracture (HR_adj_ 1.55, 95% CI: 1.42-1.68), hip fracture (HR_adj_ 1.39, 95% CI: 1.21-1.60), vertebral fractures (HR_adj_ 1.97, 95% CI: 1.63-2.37), humerus fractures (HR_adj_ 1.91, 95% CI: 1.61-2.26), and forearm fractures (HR_adj_ 1.24, 95% CI: 1.04-1.47), when compared to matched controls. When stratified by sex, results were similar between males and females (Table 2).

**Table 2.** Association between iron overload and major osteoporotic fracture sites, stratified by sex

	Patients with iron overload			Matched controls			Risk of fracture for cases vs matched controls	
	# fractures	PY	IR/1000py	# fractures	PY	IR/1000py	Unadjusted HR (95% CI)	Adjusted*^a^* HR (95% CI)
Any fracture								
All patients	775	93 816	8.26	3757	795 802	4.72	1.82 (1.68-1.97)	1.55 (1.42-1.68)
Females	428	57 008	11.60	2379	447 294	6.83	1.82 (1.63-2.02)	1.55 (1.39-1.73)
Males	347	36 808	6.09	1378	348 508	3.08	2.02 (1.79-2.28)	1.68 (1.48-1.91)
Hip fracture								
All patients	258	93 816	2.75	1425	795 802	1.79	1.63 (1.43-1.87)	1.39 (1.21-1.60)
Females	137	57 008	3.72	853	447 294	2.45	1.68 (1.40-2.02)	1.41 (1.17-1.71)
Males	121	36 808	2.12	572	348 508	1.28	1.71 (1.4-2.09)	1.47 (1.19-1.81)
Clinical vertebral fracture								
All patients	169	93 816	1.80	540	795 802	0.68	2.58 (2.16-3.09)	1.97 (1.63-2.37)
Females	83	57 008	2.25	298	447 294	0.86	2.78 (2.17-3.56)	2.09 (1.61-2.71)
Males	86	36 808	1.51	242	348 508	0.54	2.51 (1.94-3.26)	1.92 (1.47-2.52)
Humerus fracture								
All patients	184	93 816	1.96	726	795 802	0.91	2.28 (1.94-2.69)	1.91 (1.61-2.26)
Females	103	57 008	2.80	451	447 294	1.29	2.31 (1.86-2.87)	2.00 (1.60-2.50)
Males	81	36 808	1.42	275	348 508	0.62	2.47 (1.93-3.16)	1.94 (1.49-2.53)
Forearm fracture								
All patients	166	93 816	1.77	1071	795 802	1.35	1.34 (1.13-1.58)	1.24 (1.04-1.47)
Females	105	57 008	2.85	779	447 294	2.24	1.33 (1.08-1.64)	1.22 (0.99-1.51)
Males	61	36 808	1.07	292	348 508	0.65	1.65 (1.24-2.20)	1.52 (1.13-2.04)

In all analyses, the reference group is the matched control patients. Thus, overall we assess all patients with elevated serum ferritin or an eligible diagnosis compared to matched controls. In the sex-specific analysis, female patients with elevated serum ferritin or an eligible diagnosis are compared to the matched female controls, and male patients are compared to male matched controls.

Abbreviations: CI, confidence interval; HR, hazard ratio; IR, incidence rate; PY, person-years.

^*a*^Adjusted for variables with a standardized mean difference >0.1 at baseline (Table 1): smoking status and alcohol consumption at baseline; a history of cancer, chronic kidney disease, liver disease; and use of angiotensin-converting enzyme inhibitors, beta-blockers, diuretics, or oral glucocorticoids in the 6 months prior to baseline.

### Sensitivity Analysis Stratifying by Serum Ferritin Values

The baseline characteristics of patients stratified by laboratory-ascertained iron overload at baseline (ie, a serum ferritin value >1000ug/L vs diagnosis code without elevated ferritin) are provided in Table 3. We identified 13 510 patients with serum ferritin >1000ug/L at baseline and 6754 matched patients without, who were subsequently matched to 113 700 and 79 256 controls, respectively. A breakdown of the inclusion criteria based on the individual diagnosis codes is provided in Table 4, where it is notable that 87.6% of patients entering with a diagnosis code (n = 5914) entered with a diagnosis of hemochromatosis (n = 4288, 63.5%) or beta trait thalassemia (n = 1626, 24.1%). When comparing the baseline characteristics between patients with high serum ferritin values to the matched controls, similar patterns to the overall cohort were observed regarding the balance of covariates. Patients with serum ferritin >1000ug/L at baseline were more often current smokers (23.4% vs 18.2%, SMD 0.27), were current alcohol drinkers (75.7% vs 64.9%, SMD 0.29), had a history of cancer diagnosis (29.7% vs 16.6%, SMD 0.31) or liver disease (8.6% vs 2.2%, SMD 0.29), and had use of diuretics (31.9% vs 20.1%, SMD 0.27) and oral glucocorticoids (17.9% vs 9.7%, SMD 0.24) when compared to matched controls, respectively. Among patients entering the cohort with a diagnosis code but without serum ferritin >1000ug/L, there was greater imbalance of covariates at baseline. Additionally, patients were younger (mean age 47 years) and there was a higher proportion of females (48.7%) when compared to the patients with high serum ferritin (mean age 63 and 34.3% female).

**Table 3.** Baseline characteristics among patients entering the study with serum ferritin values >1000 µg/L at baseline and the matched controls

	Patients with serum ferritin >1000 μg/L at baseline*^a^*	Matched controls	SMD	Patients with eligible diagnosis code at baseline*^b^*	Matched controls	SMD
	(n = 13 510)	(n = 113 700)		(n = 6754)	(n = 79 256)	
Mean age in years (SD)	63.2 (15.1)	62.1 (15.6)	0.08	47.0 (17.0)	51.4 (19.9	0.24
Female*^d^*	4630 (34.3)	40 034 (35.2)	0.02	3292 (48.7)	41 511 (52.4)	0.07
Mean follow-up in years (SD)	3.9 (3.6)	4.1 (3.6)	0.07	6.3 (3.6)	4.3 (3.8)	0.56
Ferritin value available	13 510 (100)	3679 (3.2)	7.73	2444 (36.2)	1934 (2.4)	0.94
Ferritin *μ*g/L [mean (SD)]	1731 (1440)	153 (249)	−1.52	317 (268)	119 (155)	−0.91
BMI (closest to index date)						
Mean (SD)	27.1 (5.8)	27.6 (5.7)	0.09	27.2 (6.1)	26.9 (5.8)	0.05
Missing (%)	991 (7.3)	15 694 (15.6)		1563 (23.1)	12.542 (15.8)	
Smoking status (closest to index date) (%)			0.27			0.36
Current	3159 (23.4)	20 715 (18.2)		1108 (16.4)	16 178 (20.4)	
Former	4324 (32.0)	32 581 (28.7)		1271 (18.8)	17 229 (21.7)	
Never smoker	5902 (43.7)	55 289 (48.6)		3349 (49.6)	42 176 (21.7)	
Missing	125 (0.9)	16 314 (14.3)		1026 (15.2)	3673 (4.6)	
Alcohol consumption (closest to index date) (%)			0.29			0.25
Current	10 229 (75.7)	73 816 (64.9)		3752 (55.6)	48 134 (60.7)	
Former	617 (4.6)	3849 (3.4)		145 (2.1)	2013 (2.5)	
Never	5902 (43.7)	19 721 (17.3)		1037 (15.4)	15 618 (20.4)	
Missing	1012 (7.5)	16 314 (14.3)		1820 (26.9)	13 491 (17.0)	
Covariates (ever before index date) (%)						
Aplastic anemia	45 (0.3)	14 (0.0)	0.08	<7 (0.1)	7 (0.0)	0.04
Cancer diagnosis	4006 (29.7)	18 838 (16.6)	0.31	654 (9.7)	9441 (11.9)	0.07
Chronic kidney disease	498 (3.7)	1632 (1.4)	0.14	40 (0.6)	758 (1.0)	0.04
Hemolytic pernicious	195 (1.4)	676 (0.6)	0.08	41 (0.6)	402 (0.5)	0.01
Inflammatory diseases*^c^*	375 (2.8)	2030 (1.8)	0.07	97 (1.4)	1156 (1.5)	0.01
Thyroid disease	958 (7.1)	7615 (6.7)	0.02	388 (5.7)	4482 (5.7)	0.01
Ulcers	604 (4.5)	3633 (3.2)	0.07	123 (1.8)	1669 (2.1)	0.02
Pregnancy*^d^*	65 (0.5)	495 (0.4)	0.01	71 (1.1)	743 (0.9)	0.01
Hypogonadism	22 (0.2)	152 (0.1)	0.01	9 (0.1)	44 (0.1)	0.03
Osteoporosis	588 (4.4)	3632 (3.2)	0.06	122 (1.8)	2172 (2.7)	0.06
Liver disease	1162 (8.6)	2501 (2.2)	0.29	258 (3.8)	1110 (1.4)	0.15
Endocrine disease	55 (0.4)	231 (0.2)	0.04	13 (0.2)	141 (0.2)	0.01
Prior fracture*^e^*	1611 (11.9)	10 788 (9.5)	0.08	551 (8.2)	6945 (8.8)	0.02
Medication (within 6 months before index date) (%)						
ACE inhibitors	4194 (31.0)	27 072 (23.8)	0.16	1014 (15.0)	12 935 (16.3)	0.04
Antihypertensives	505 (3.7)	2840 (2.5)	0.07	79 (1.2)	1306 (1.6)	0.04
Beta-blockers	3081 (22.8)	16 083 (14.1)	0.23	589 (8.7)	7751 (9.8)	0.04
Calcium channel blockers	2577 (19.1)	17 354 (15.3)	0.10	582 (8.6)	8043 (10.1)	0.05
Diuretics	4310 (31.9)	22 846 (20.1)	0.27	851 (12.6)	11 363 (14.3)	0.05
Oral glucocorticoids	2417 (17.9)	11 033 (9.7)	0.24	558 (8.3)	6264 (7.9)	0.01
Bisphosphonates	612 (4.5)	3266 (2.9)	0.09	89 (1.3)	1936 (2.4)	0.08
NIADs	1013 (7.5)	8860 (7.8)	0.01	233 (3.4)	3882 (4.9)	0.07
Insulin	322 (2.4)	2290 (2.0)	0.03	55 (0.8)	1135 (1.4)	0.06

Unless otherwise indicated, all values are the number and column proportions.

Abbreviations: BMI, body mass index; NIADs, non-insulin antidiabetic drugs; SMD, standardized mean difference (calculated as Cohen's *d*).

^*a*^Patients identified at baseline based on the presence of having lab value with serum ferritin >1000 µg/L, regardless of the presence of an eligible diagnosis code.

^*b*^Patients identified at baseline based on an eligible diagnosis code [Supplementary Table S1 (23)] but without evidence of elevated serum ferritin values.

^*c*^Includes a diagnosis of rheumatoid arthritis, psoriatic arthritis, inflammatory bowel disease, or other unspecified inflammatory disease.

^*d*^Pregnancy was identified based on diagnosis codes and identified within the 9 months prior to the index date.

^*e*^Prior fractures were those occurring >365 days before the index date.

**Table 4.** Overview of the number of patients included in the study with an eligible diagnosis code (note counts <7 are suppressed for patient privacy)

Code	Name	Count
D104500	Beta trait thalassemia	1626
C350000	Hemochromatosis	4288
D104.00	Thalassemia	583
D104811	Beta thalassemia	136
D104211	Sickle-cell thalassemia	37
D104z00	Thalassemia NOS	22
C350.00	Disorders of iron metabolism	36
D104700	Beta major thalassemia	<7
D104000	Thalassemia major NEC	<7
D104600	Beta intermedia thalassemia	<7
D104200	Thalassemia with hemoglobin S disease	<7
D104900	Delta-beta thalassemia	10
C350z00	Disorder of iron metabolism NOS	<7
C350y00	Other specified disorder of iron metabolism	8
D104011	Thalassemia major—Cooley's anemia	<7

Table 5 presents the risk estimates for the association between iron overload and osteoporotic fracture risk, stratified by the presence of confirmed laboratory serum ferritin >1000ug/L at baseline. Among patients with serum ferritin >1000ug/L, there was a significantly increased risk of any osteoporotic fracture (HR_adj_ 1.91, 95% CI: 1.73-2.10), while there was no association observed among patients entering the cohort based on an eligible diagnosis code (HR_adj_ 0.99, 95% CI: 0.84-1.16) compared to matched controls. When stratified by fracture site, patients with serum ferritin >1000ug/L had an increased risk of hip fracture (HR_adj_ 1.77, 95% CI: 1.51-2.08), vertebral fractures (HR_adj_ 2.51, 95% CI: 2.01-3.12), humerus fractures (HR_adj_ 2.41, 95% CI: 1.96-2.95), and forearm fractures (HR_adj_ 1.31, 95% CI: 1.05-1.64). No risks were identified among patients entering based on an eligible diagnosis code without a recent laboratory serum ferritin test indicating a value >1000ug/L at any fracture site. Risk estimates stratified by biologic sex are provided in Table 6. No sex-specific differences were identified.

**Table 5.** Association between iron overload and major osteoporotic fracture sites, stratified by evidence of iron overload (serum ferritin >1000ug/L) at cohort entry date

	Patients with iron overload			Matched controls			Risk of fracture for cases vs matched controls	
	# fractures	PY	IR/1000PY	# fractures	PY	IR/1000py	Unadjusted HR (95% CI)	Adjusted HR*^bc^* (95% CI)
Any fracture								
Ferritin >1000 *μ*g/L	593	51 205	11.60	2279	460 497	4.95	2.31 (2.11-2.54)	1.91 (1.73-2.10)
Eligible diagnosis *^a^*	182	42 611	4.27	1478	335 305	4.41	1.01 (0.87-1.18)	0.99 (0.84-1.16)
Hip fracture								
Ferritin >1000 *μ*g/L	221	51 205	4.32	909	460 497	1.97	2.14 (1.84-2.49)	1.77 (1.51-2.08)
Eligible diagnosis*^a^*	37	42 611	0.87	516	335 305	1.54	0.63 (0.46-0.87)	0.68 (0.49-0.95)
Clinical vertebral fracture								
Ferritin >1000 *μ*g/L	135	51 205	2.64	333	460 497	0.72	3.47 (2.83-4.25)	2.51 (2.01-3.12)
Eligible diagnosis*^a^*	34	42 611	0.79	207	335 305	0.62	1.28 (0.89-1.84)	1.04 (0.69-1.56)
Humerus fracture								
Ferritin >1000 *μ*g/L	141	51 205	2.75	438	460 497	0.95	2.95 (2.44-3.57)	2.41 (1.96-2.95)
Eligible diagnosis*^a^*	43	42 611	1.01	288	335 305	0.86	1.25 (0.92-1.71)	1.20 (0.86-1.67)
Forearm fracture								
Ferritin >1000 *μ*g/L	98	51 205	1.91	602	460 497	1.31	1.42 (1.15-1.77)	1.31 (1.05-1.64)
Eligible diagnosis*^a^*	68	42 611	1.60	469	335 305	1.40	1.16 (0.91-1.49)	1.17 (0.89-1.53)

In all analyses, the reference group is the matched control patients.

Abbreviations: CI, confidence interval; HR, hazard ratio; IR, incidence rate; PY, person-years.

^*a*^Patients identified at baseline based on an eligible diagnosis code [Supplementary Table S1 (23)] but without evidence of elevated serum ferritin values.

^*b*^Models for iron overload patients adjusted for smoking status and alcohol consumption at baseline and a history of cancer, liver disease, and use of angiotensin-converting enzyme inhibitors, beta-blockers, diuretics, and oral glucocorticoids in the 6 months prior to baseline.

^*c*^Models for patients without iron overload adjusted for age, smoking status, and alcohol consumption at baseline, history of liver disease.

**Table 6.** Sex-specific association between iron overload and major osteoporotic fracture sites, stratified by evidence of iron overload (serum ferritin >1000ug/L) at cohort entry date

	Males Adjusted HR (95% CI)*^b^*	Females Adjusted HR (95% CI)*^a^*
Any fracture	1.68 (1.48-1.91)	1.55 (1.39-1.73)
Ferritin >1000 *μ*g/L	2.11 (1.82-2.44)	1.93 (1.68-2.21)
Eligible diagnosis*^a^*	0.96 (0.74-1.26)	1.06 (0.88-1.29)
Hip fracture	1.47 (1.19-1.81)	1.41 (1.17-1.71)
Ferritin >1000 *μ*g/L	1.85 (1.46-2.33)	1.86 (1.49-2.32)
Eligible diagnosis*^a^*	0.62 (0.36-1.05)	0.73 (0.49-1.10)
Vertebral fracture	1.92 (1.47-2.52)	2.09 (1.61-2.71)
Ferritin >1000 *μ*g/L	2.45 (1.79-3.36)	2.75 (2.02-3.75)
Eligible diagnosis*^a^*	1.11 (0.63-1.95)	1.04 (0.61-1.78)
Humerus fracture	1.94 (1.49-2.53)	2.00 (1.60-2.50)
Ferritin >1000 *μ*g/L	2.71 (2.00-3.66)	2.41 (1.82-3.20)
Eligible diagnosis*^a^*	0.78 (0.41-1.46)	1.48 (1.03-2.12)
Forearm fracture	1.52 (1.13-2.04)	1.22 (0.99-1.51)
Ferritin >1000 *μ*g/L	1.67 (1.16-2.41)	1.29 (0.97-1.73)
Eligible diagnosis*^a^*	1.41 (0.85-2.32)	1.21 (0.88-1.66)

In all analyses, the reference group is the matched control patients.

Abbreviations: CI, confidence interval; HR, hazard ratio.

^*a*^Patients identified at baseline based on an eligible diagnosis code [Supplementary Table S1 (23)] but without evidence of elevated serum ferritin values.

^*b*^Models for iron overload patients adjusted for smoking status and alcohol consumption at baseline and a history of cancer, liver disease, and use of angiotensin-converting enzyme inhibitors, beta-blockers, diuretics, and oral glucocorticoids in the 6 months prior to baseline.

The results from the post hoc analysis are presented in Table 7. After adjusting for imbalanced confounding variables, there was a significant risk of any osteoporotic fracture among patients with the presence of confirmed laboratory serum ferritin >1000ug/L compared to patients with a diagnosis of an iron overloading disorder without confirmed laboratory serum ferritin >1000ug/L at baseline (HR_adj_ 1.57, 95% CI: 1.31-1.88).

**Table 7.** Association between serum ferritin >1000 µg/L and major osteoporotic fracture sites, compared to patients with an eligible diagnosis code for an iron overloading disorder without laboratory-confirmed serum ferritin >1000 µg/L

	# fractures	PY	IR/1000PY	Unadjusted HR (95% CI)	Adjusted HR (95% CI)*^a^*
Eligible diagnosis	182	42 611	4.27	Reference	Reference
Ferritin >1000 µg/L	593	51 205	11.60	2.67 (2.26-3.15)	1.57 (1.31-1.88)

Abbreviations: CI, confidence interval; HR, hazards ratio; IR, incidence rate; PY, person-years.

^*a*^Adjusted for age, sex, smoking status, alcohol use, cancer diagnosis, chronic kidney disease, liver disease, prior fractures, ace inhibitors, beta blockers, calcium channel blockers, diuretics, oral glucocorticoids, non-insulin antidiabetic drugs (NIADs).

## Discussion

Given the lack of data regarding the impact of iron overload on fracture risk, we analyzed the fracture risk in individuals with iron overload in a large population-based study. We found that patients with iron overload (identified as those with serum ferritin >1000ug/L or a diagnosis of an iron overloading disorder) had a significant 55% overall increased risk of any major osteoporotic fracture. The risk was highest for clinical vertebral and humerus fracture with an almost 2-fold increased risk observed. Importantly, the increased fracture risk was driven by the elevated risk among patients with laboratory-ascertained iron overload, as measured by having a serum ferritin value above 1000ug/L, while there was no elevated risk among patients with a diagnosis code for an iron overloading disorder in the absence of elevated serum ferritin. When comparing the fracture risk for an osteoporotic fracture among only patients with iron overload, those with a serum ferritin value above 1000ug/L had a 57% increased risk of fracture compared to those with a diagnosis of an iron overloading disorder but without elevated serum ferritin. Thus, the results of this large cohort study highlight the importance of managing fracture risk among patients with high iron overload and should raise awareness of skeletal fragility in patients with iron overload.

Our results are in line with smaller-scale studies indicating an increased fracture risk in patients with *HFE* HH (7, 25) and iron-loading anemias (11). A previous study with 306 patients with *HFE* HH indicated a 6-fold higher prevalence of osteoporosis, which was associated with a 1.7-fold nonsignificant higher rate of fractures at the wrist and spine but not the hip (7). This study also showed that osteoporosis was positively associated with iron overload and disease duration and that the odds ratio for wrist and spine fractures increased 3-fold when dividing patients into high (>1000 µg/L ferritin) and low (<1000 µg/L ferritin) ferritin groups (7). Similar results were found in a more recent study that looked into “bone fragility,” a composite measure of T-score and the occurrence of fragility fractures, in 93 patients with *HFE* HH, highlighting the close association of bone fragility with the extent of iron overload, disease duration, and ferritin concentrations (25). It is of interest that the highest risk for fractures was reported at the wrist and spine in the Richette et al study, similar to our findings. The reason for this is unclear but may suggest site-specific differences of iron effects on bone microarchitecture and fracture resilience. Earlier studies have shown no difference between BMD at the femoral neck vs the spine in patients with *HFE* HH, suggesting that bone quality may account more for the increased fracture risk at the spine and wrist (15). Similarly, pronounced effects on the BMD of the lumbar spine were also observed in sickle cell disease and thalassemia (26-29). Thus, more detailed prospective studies are necessary to clarify these site-specific vulnerabilities of bone to iron overload. One should also look into physiological stresses and forces as well as causes of trauma and fracture.

Interestingly, a previous study using a large population-based cohort from the UK General Practice Research Database showed no higher fracture risk among the 501 patients with hemochromatosis (30). This study included patients based on the recorded diagnosis code of hemochromatosis only. This compares to our results when stratifying only by the diagnosis codes for hemochromatosis, sickle cell anemia, and thalassemia major. Indeed, our analysis identified that the fracture risk was restricted to patients with high serum ferritin levels, both when compared to controls with iron overloading disorders and when compared to patients with a diagnosis for an eligible iron overloading condition. This is also supported by recent findings in mice, in which bone cell-specific knockout of *HFE* was not associated with bone disease (31).

Serum ferritin levels above 1000 µg/L have been defined as a critical cut-off for patients with homozygous *HFE* C282Y mutations (32). Above this threshold, patients are at particularly high risk of developing symptoms associated with the *HFE* HH, such as liver disease, fatigue, and osteoarthritis based on the use of arthritis medications (32). Thus, as we were unable to confirm HFE C282Y mutations in our database, we opted to stratify by serum ferritin, which is more indicative of iron overload. In our analysis, the risk of hip fracture, clinical vertebral, and humerus fracture was substantially higher among those with serum ferritin levels above 1000 µg/L compared to those without elevated serum ferritin. Additionally, the post hoc analysis identified that elevated serum ferritin was associated with a 57% increased risk of fracture compared to having a diagnosis of an iron overloading disorder without having elevated iron levels. Cumulatively, these results underline the clinical importance of iron overload on fracture risk and not the underlying diseases per se, as patients had comparable baseline characteristics and a time-varying confounder was applied.

## Strengths and Limitations

This is the largest population-based study on the association between iron overload and fractures to date. The use of the IMRD-UK enabled us to identify serum ferritin values rather than rely on diagnosis codes alone. Indeed, the majority of patients included in the analysis were identified based on laboratory values rather than diagnosis codes. The increased sample size enabled us to stratify based on the presence of laboratory-ascertained iron levels, ruling out misclassification that may happen when only diagnosis codes are present. Additionally, due to the robust cohort size, we could adjust for relevant confounders, including diagnoses, medication use, and alcohol consumption.

However, despite the strengths of the database, our study may have potential limitations. First, while electronic healthcare records are rich sources for population-based research, it is important to note that there is the possibility of exposure misclassification among our matched controls. Indeed, it is possible that the controls may have elevated iron levels but had not received a blood test from their treating physician. Indeed, only 2.9% of controls had a serum ferritin value available at baseline. However, in the event of exposure misclassification in our control patients, we would expect this to result in a bias toward the null hypothesis, as it would result in the misclassified controls having a higher fracture risk. As we identified a significant increased fracture risk, we do not expect this limitation to negate the study results.

An additional limitation was that we did not assess the duration of iron overload on fracture risk. Thus, it is possible that patients could enter the cohort with a single elevated serum ferritin value, which may not be a result of systemic iron overload. We did exclude patients with recent iron supplementation to minimize this risk of medication-induced temporary iron overload; however, future analyses should aim to investigate the relationship between duration and severity of iron overload on fracture risk. We also did not adjust for the use of iron supplementation during follow-up as this was expected to fall on the causal pathway (ie, the use of iron supplementation would be determined based on the presence of existing iron overload, which would then influence fracture risk), making adjustment in the model inappropriate.

We also did not assess the impact of changes in serum ferritin levels during follow-up—for example, identifying the impact of reducing serum ferritin <1000 µg/L during follow-up. Therefore, future work to determine if decreasing ferritin is associated with a lower risk of fracture compared to a steady state of high iron levels among patients with laboratory ascertained serum ferritin values >1000 µg/L would be of high interest. Similarly, we did not evaluate the impact of treatment strategies as our comparator group was nonpatients. Clinically, the most important therapy is phlebotomy with a target serum ferritin of 50 ng/mL and normal transferrin saturation. Genetic therapy for HFE hemochromatosis, which goes along with deficiency of the iron regulatory hormone hepcidin, is not yet state-of-the-art or in clinical practice. The impact of these therapies on fracture risk reduction is of interest for future work.

Due to limitations in the data, we were not able to adjust or correct for inflammation status due to too few patients having C-reactive protein measurements and the lack of availability of leukocyte count or procalcitonin in our primary care. We acknowledge that serum ferritin can be elevated during periods of infection and inflammation. To avoid misclassifying patients, we restricted the inclusion to patients with serum ferritin >1000 µg/L in the absence of an eligible diagnosis code. Indeed, while serum ferritin >200/300 µg/L is often used to identify iron overload, we acknowledge that values ranging between 300 and 1000 µg/L can be due to infections or other conditions. Thus, while we increased specificity for iron overload, it is possible that patients in our matched controls had more moderate iron overload (ie, serum ferritin between 300 and 1000 µg/L). In this case, we would expect the overall effect to be attenuated, leading to a reduced effect size (ie, toward the null hypothesis). Additionally, we expect the patients with severe inflammatory conditions leading to serum ferritin >1000 µg/L to be treated in ambulatory, not primary care, settings.

While previous studies have shown the validity of fracture coding within the UK General Practice data (21, 24), we acknowledge that there is a potential for outcome misclassification regarding vertebral fractures. We are only able to include vertebral fractures that were brought to clinical attention and received a diagnosis. Not all vertebral fractures are symptomatic, and therefore it is likely that we have underestimated the incidence of vertebral fractures. Moreover, it is possible the date of the fracture is misclassified as it may have not been detected immediately. However, we do not expect differences in the misclassification rate between the iron cases and the matched controls since the evidence on the association between iron overload and vertebral fractures is not well established.

Finally, while we were able to adjust for measured confounders that were imbalanced at baseline, we could not assess unmeasured and inherent confounding factors, such as the presence of genetic variants, dietary aspects, BMD, and exercise. However, given the strength of the observed association, particularly among patients with laboratory-ascertained iron overload, after matching and adjusting for measured confounders we do not expect that unmeasured confounding would nullify the observed results.

## Conclusions and Clinical Implications

Overall, this large population-based study identified that patients with iron overload have a 55% increased risk for fracture, particularly for clinical vertebral and humerus fractures where the risk almost doubled when compared to matched patients without iron overload. Among patients with serum ferritin >1000 µg/L, the risk increased substantially, with patients having a 91% increased risk of any fracture compared to matched controls and 57% increased risk when compared to patients with a diagnosis of an iron overloading condition but without laboratory-confirmed high serum ferritin. There were no significant differences between males and females and no elevated fracture risk among patients with a diagnosis of an iron overloading condition but without laboratory-confirmed high serum ferritin.

Cumulatively, our study results confirm the most recent recommendation by the European Association for the Study of the Liver for clinicians to evaluate extrahepatic manifestations (including heart failure and diabetes as well as bone health and fracture risk) in patients with hemochromatosis (33). While serum ferritin levels >200 µg/L in premenopausal women and >300 µg/L in men and postmenopausal women may indicate primary iron overload due to hemochromatosis, especially when associated with high transferrin saturation and evidence of liver disease (34), clinicians should still be aware at these levels and initiate clinical examination including laboratory ferritin levels, genetic testing for HFE or non-HFE hemochromatosis, and iron-magnet resonance imaging; however, our results suggest that the main risk group for fracture risk is among those patients with high iron burden, namely those with serum ferritin >1000ug/L. Among these patients, lifestyle modifications, fall reduction strategies, regular phlebotomy or blood donations to reduce excessive iron deposition, and osteoporosis treatment are important management strategies (6, 34).

Thus, the main clinical message from our findings is that clinicians should consider iron overloading as a risk factor for fracture. Importantly, among high-risk patients presenting with serum ferritin values exceeding 1000 µg/L, osteoporosis screening and treatment strategies should be initiated in accordance with the guidelines for patients with hepatic disease.

## Data Availability

Access to the data used in this study were purchased from IQVIA and cannot be shared publicly or shared with other research without review and consent from IQVIA due to the sensitive nature of the patient data. However, interested persons can contact the corresponding author regarding the data for inquiry and data and statistical coding.
